# Role of magnetic resonance urography in diagnosis of duplex renal system: Our initial experience at a tertiary care institute

**DOI:** 10.4103/0970-1591.45537

**Published:** 2009

**Authors:** Milind P. Joshi, Heemanshi S. Shah, Sandesh V. Parelkar, Amit A. Agrawal, Beejal Sanghvi

**Affiliations:** Department of Paediatric Surgery, KEM Hospital, Mumbai, India

**Keywords:** Duplex renal system, intravenous urography, magnetic resonance urography, micturating cystourethrography, ultrasound

## Abstract

**Aim:**

To determine diagnostic value of magnetic resonance urography in cases of duplex renal system.

**Method:**

Twenty cases between five month to nine years with suspected or known duplex renal system were evaluated by ultrasound (USG), micturating cystourethrography (MCU), intravenous urography (IVU) and magnetic resonance urography (MRU). The findings of these diagnostic imaging studies were then compared with each other and against the results of final diagnosis established at surgery.

**Results:**

Duplex renal system could be identified in two of these cases on USG, was diagnosed in four in IVU and could be diagnosed in all cases with MRU.

**Conclusion:**

MRU is superior and far accurate than IVU, MCU and USG in diagnosing duplex renal system.

## INTRODUCTION

Conventional radiological investigations are commonly being used for the diagnosis of duplex renal system. However, each of them has limitations and hence multimodality workup is required in their evaluation.

It may be possible to avoid it with its associated cost, if similar information were available from a single imaging modality which will reduce the time and will give better diagnosis using magnetic resonance urography (MRU) for its diagnosis. This article describes our initial experience using MRU for the diagnosis of duplex renal system and comparing its findings with other conventional radiological investigations.

The concept of MRU has been introduced since 1986 by Henning *et al.*[1–3] and is being used since then with its various modifications as per the indication of study.

The purpose of the study was to confirm the efficacy of MRU and comparing its findings with other radiological investigations.

## MATERIALS AND METHODS

This was a prospective study done over a period of one year from 2006 to 2007. At the outset of the study the patients with diagnosed or suspected duplex renal anomalies on routine radiological investigations or those not picked on conventional investigations as intravenous urography (IVU), micturating cystourethrography (MCU), ultrasound (USG) and nuclear scintigraphy and having persitant urogical symptoms underwent MRU using gadolinium contrast after obtaining proper consent for the procedure. IVU, MCU were performed using standard protocols of the procedure and USG was done in all the cases. Nuclear scintigraphy study was done using technetium T99 labeled diethylenetriaminopentaacetic acid (DTPA) and dimercaptosuccinic acid (DMSA) with lasix at 10 min of study.

MRU was done using heavily T2-weighted images, contrast enhanced T1-weighted MR sequences and maximum intensity projection (MIP) after proper hydration in 1.5T MR scanner imaging.

Very young and uncooperative patients were given oral sedation. These patients were given as per body weight to maintain proper hydration. Intravenous gadolinium contrast was used for the study for image acquisition using abdominal or body coil, with patient in supine position and coil positioned over upper abdomen and centered on kidneys. After initial localizing images were obtained in the following sequences:

T2 HASTE single slice

T2 HASTE multislice

3D GRE T1

TRUFI 2D

FL2D 80

TSE FS T2

Post contrast images in T1 sequences were obtained.

## RESULTS

Total twenty cases were subjected for MRU after they were either diagnosed to have duplex on conventional radiological investigations or were suspected to be having duplex renal system and routine radiological investigations failed to pick the condition.

USG was suspicious in two cases and IVU showed four cases to be having duplex renal system including these two cases. MRU successfully picked up duplex moiety in either of the side in all of these patients. Fourteen were female patients and rests were male. Three patients had bilateral duplex renal system. Ten patients underwent upper pole heminephrectomy for nonfunctioning upper moiety. One patient had both the moieties functioning and hence underwent upper pole ureteric reimplantation for the refluxing moiety. Two patients had pathological single unit on one side and normally functioning duplex unit on the other side and underwent nephrectomty of the pathological single unit of the opposite side. Two patients are under regular follow up and are asymptomatic and on chemoprophylaxis and doing well and two patients did not turn up for further follow up. One patient had unilateral duplex with ureterocele of the upper mioty which was normally functioning and incision of uereterocele was done. We compared the sensitivity and specificity of the IVU and MRU in the diagnosis of the duplex system and compared it with operative findings. MRU showed the sensitivity and specificity of 100% as compared to only forty percent sensitivity of the IVU. But specificity of IVU was 100% here.

## DISCUSSION

Duplication is the most common congenital anomaly in the urinary tract, a 0.7% incidence in one series.[4] During initial screening, the diagnosis of duplex system may be possible by ultrasound evaluation. It can also detect associated renal dysplasia, hydronephrotic changes of upper or lower moity and associated ureterocele if present.[5] However, it is highly observer dependent and many times duplex anomaly can be missed and also fails to identify duplex mioties when associated with hydroureters.

MCU can diagnose associated refluxing unit in cases of duplex moity and ureterocele however as a single investigation cannot diagnose duplex system.

IVU which is considered as a standard investigation for morphological assessment of renal parenchyma fails to identify nonfunctioning upper moieties of duplex system.

Abnormal alignment of upper pole calices of lower moity because of pressure effect of dysplastic upper moity can be seen on IVU which may give clue to the diagnosis.

Renal scintigraphy is necessary to know the differential functional status of the duplex moieties which influences the treatment. It however gives poor anatomical delineation.

In 1996, Pearlman *et al.* described the concept of computed tomographic urography (CTU) as a diagnostic method in renal diseases. Multislice computed tomography (CT) with contrast and 3D reconstruction also gives excellent anatomical details but has high radiation exposure and risk of allergy to the contrast.[6]

We found that, MR urography as a single investigation gives excellent anatomical description of the duplex renal moieties even when they are nonfunctioning. MRU is a noninvasive examination method that does not entail ionizing radiations and does not require iodinated contrast as in IVU or CTU and safer in children. It is excellent in identifying nonfunctioning or poorly functioning duplex system.

**Figure 1:**
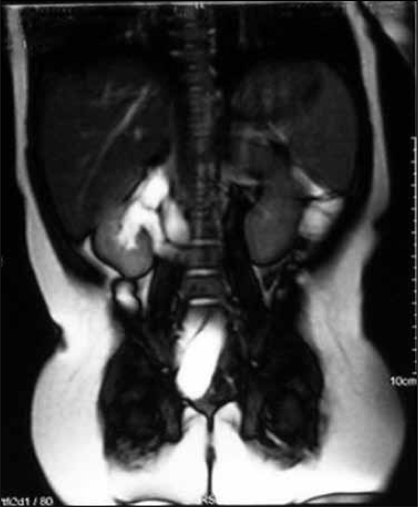
T2 weighted noncontrast images showing dilated ureters of both the moities

**Figure 2:**
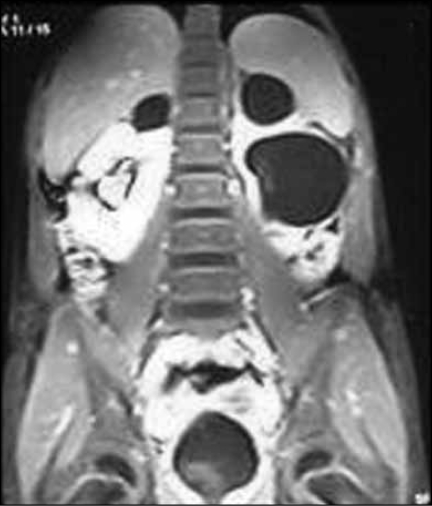
Contrast enhanced T1 weighted image showing normally functioning right lower moity

**Figure 3:**
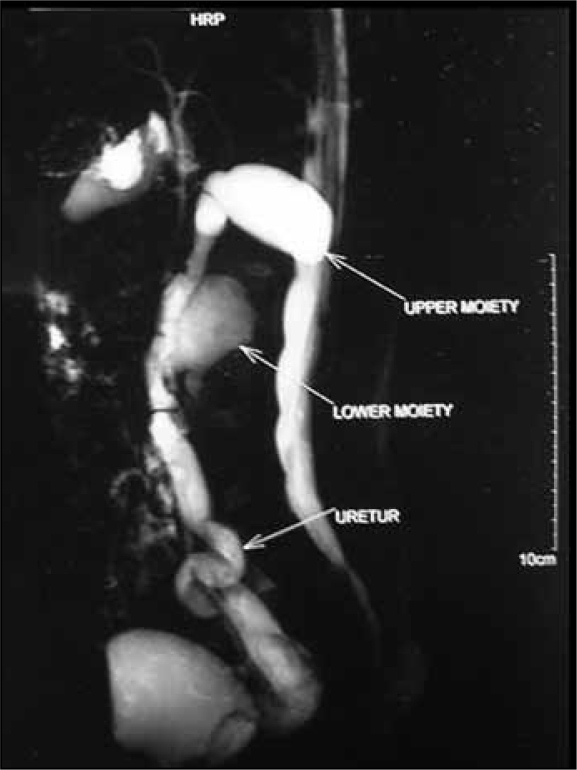
Duplex moities with their respective ureter and common distal ureteric sheath

The heavily weighted T2 images best pick up the dilated non functioning moieties using static fluid as hyperintence images [Figure 1]. This technique does not require contrast.[7] The T1-weighted images pick up nondilated and functioning unit as hyperintence [Figure 2]. Development of faster sequences, the GRE-T1 weighted contrast sequences provided the opportunity for faster examination. It is possible to obtain IVU -like images by employing maximum intensity projection (MIP) method on heavily T2 weighted images and contrast enhanced T1 images[8] [Figure 3]. In our study, however sensitivity and specificity could be calculated in only those cases where operative intervention was required and hence this high value may look unreal.

But there are studies where this is calculated and found to be almost 99% sensitive and more than ninety five percent specific for the diagnosis of the duplex renal system.[5,6]

We attribute the reasons for failure of IVU to diagnose the duplex system where operative intervention was not required to presence of ectopic moity superimposed on the bony structures and could not be seen in IVU in three cases, borderline functioning in these same cases, poor patient preparation in another two cases and where the pressure from the dilated lower moity was obstructing the drainage as well as compromising the perfusion of the duplex moity and hence was appearing like non functioning moity on IVU. In one case, the anatomical arrangement of the calyses was so close to each other that on IVU they resembled as calyses of the single unit and misinterpreted by the radiologist and only MRU could detect the duplex in this.

It is obvious that conventional radiology cannot singly pick up duplex renal anomaly and hence there is no single gold standard conventional investigation for its diagnosis and here the role of MRU appears to be superior to all conventional investigations and also gives better anatomical and functional accuracy in diagnosing duplex system which surpasses its disadvantages of cost, time, and need of sedation for the procedure.[2,5,9,10]

Moreover, the time of examination for MRU is lesser than for scintigraphy done for quantification of function.[11]

In our cases, MRU successfully picked up duplex renal system and in the cases where operative intervention was required, also helped in deciding the plan of management which was not possible by conventional investigations.

In our initial experience, MRU may become the single investigative modality compared to other conventional radiological investigations in cases of duplex renal system.[12–14]
